# Predictors of Definitive Treatment Interruptions of Long-Course Neoadjuvant Chemoradiotherapy in Locally Advanced Rectal Cancer

**DOI:** 10.7759/cureus.30159

**Published:** 2022-10-10

**Authors:** Lucas G Sapienza, Sreejata Raychaudhuri, Suraya K Nahlawi, Serene Ozeir, Eyad Abu-Isa

**Affiliations:** 1 Radiation Oncology, Baylor College of Medicine, Houston, USA; 2 Hematology and Oncology, University of Pittsburgh Medical Center, Pittsburgh, USA; 3 Radiology, Ascension Providence Hospital, Southfield, USA; 4 Internal Medicine, Michigan State University, East Lansing, USA; 5 Radiation Oncology, University of Michigan, Ann Arbor, USA

**Keywords:** acute toxicity, treatment interruption, neoadjuvant, radiotherapy, rectal cancer

## Abstract

Introduction

To identify predictors of definitive treatment interruptions (DTI) of the neoadjuvant long-course radiotherapy (LCRT) in locally advanced rectal cancer (LARC), and to determine their impact on clinical outcomes.

Methods

Patients with stage II-III LARC treated between 2009-2018 were retrospectively analyzed (n=101, median FU 49.5 months). Logistic regression models evaluated the impact of relevant clinical variables on grade 3 or greater (G3+) acute toxicity, definitive treatment interruption (DTI), pCR, and definitive ostomy (dOST) rates. The secondary outcomes were LRC, MFS, PFS, CSS, and OS.

Results

The incidences of grade 3 and 4 toxicities were 25.3%, and 1.1%, respectively. The most common G3+ toxicities were peri-anal dermatitis (14.7%) and diarrhea (7.4%), which were more frequent in females (*p*=0.040) and tumors close to the anal verge (*p*=0.019). In this study, 11 patients (10.9%) developed DTI, which was associated with these G3+ events (*p*<0.001). Resection occurred after 7.1 weeks (median, IQR:6.1-8.9). Downstaging occurred in 57.4% (17.8% pCR), 88% achieved negative margins and the dOST rate was 56.4%. The five-year LRC, MFS, PFS, CSS and OS were: 94.4%, 78.9%, 74.7%, 85.2% and 81.6%, respectively. DTI events did not impact any outcome. The factors associated with loco-regional failure were close/positive margins (*p*<0.001) and stage ypIII (p=0.002).

Conclusions:

Tumors close to the anal verge and female sex were associated with increased G3+ toxicity, which was predictive of DTI. The resultant partial/complete omission of the planned boost, however, dose did not increase the chance of LR. Further studies to clarify the benefit and optimal timing to deliver the boost are warranted, especially for positive margins.

## Introduction

Locally advanced rectal cancer (LARC) is better controlled with the combination of two local treatments. Influenced by the results of the CAO/ARO/AIO-94 [1,2] and NSABP R-03 studies [3], the current standard of care favors starting with radiotherapy (RT) followed by surgical resection. The most used neoadjuvant RT regimens are long course (LCRT), which delivers 25-28 fractions (1.8-2.0 Gy) with concurrent chemotherapy; and short course (SCRT), consisting of 25 Gy in 5 fractions of 5.0 Gy.

The relatively higher prescription dose utilized in the LCRT requires a more protracted delivery (five to six weeks vs. one week for SCRT). The possible increased tumor downstaging rate [4-6] comes at the expense of more acute toxicity events. These on-treatment toxicity events can reduce treatment compliance causing definitive treatment interruptions (DTI). To illustrate that, the neoadjuvant arm of the CAO/ARO/AIO-94 trial [1] reported that 27% of patients developed G3+ toxicity and 8% did not receive the total radiotherapy dose. In the same direction, 8.0%, 7.4%, and 3.0% of the patients did not receive full RT dose in the LCRT (with fluorouracil) arms of STAR-01 [7], TROG 01.04 [8], and CAO/ARO/AIO-04 [9] studies, respectively. To date, it is unclear whether these DTIs affect prognosis in LARC.

A detailed examination of the factors causing these DTIs could provide relevant insights to improve the RT technique, including indirect evidence regarding the optimal prescription dose. Based on that, we reviewed our experience with the main objectives: (i) calculate the rate of cases that developed definitive treatment interruption (DTI); (ii) analyze factors that predispose to DTI; (iii) evaluate the impact of DTI on outcomes.

## Materials and methods

Study design

All patients with a diagnosis of rectal cancer referred to the radiation oncology department between 2009 and 2018 were retrospectively screened (n=211). After applying the exclusion criteria, we included 101 consecutive patients with LARC (American Joint Committee on Cancer - AJCC stages II and III) that received long-course neoadjuvant chemoradiotherapy followed by surgical resection. The institutional review board (IRB) approved this study design and the use of patient information without individual identification (IRB number 1466256-1). This article was previously posted to the Research Square preprint server on May 17th, 2021 [10].

Treatment protocol

Radiotherapy consisted of 45 Gy (25 daily fractions of 1.8 Gy) to the pelvis plus 5.4 Gy (additional 3 fractions of 1.8 Gy) to the gross disease, including primary tumor and suspicious enlarged regional nodes. The mesorectum, presacral nodes, and internal iliac nodes were electively covered for all cases. Cases with extension to anterior pelvic structures (prostate, uterus, or bladder) had the external iliac chain prophylactically treated. Of the 22 cases with tumors involving or extending below the dentate line (defined as 2.1cm above the anal verge [11]), the bilateral inguinal lymph nodes were electively covered in 7 patients (31.8%), per radiation oncologist preference. Concurrent chemotherapy consisted of fluoropyrimidine-based (5-FU or capecitabine). Surgery was performed using the principles of total mesorectal excision (TME). Additional adjuvant chemotherapy was used after surgery per the medical oncologist's discretion.

Endpoints

The rates of grade 3 or greater (G3+) acute toxicity, definitive treatment interruption (DTI), pathological complete response (pCR: ypT0ypN0), and definitive ostomy (dOST: no stoma at the last follow-up) are the main study outcomes. Toxicity was obtained from the on-treatment weekly evaluations performed by the radiation oncologist. These were graded retrospectively according to the general guidelines of the Common Terminology Criteria for Adverse Events v4.0 [12]. The toxicity analysis was based on 95 instead of 101 cases as no toxicity description was found in the electronic medical records for six cases. The secondary study outcomes are loco-regional control (LRC), metastasis-free survival (MFS), progression-free survival (PFS; composite of locoregional failure, distant failure, or death), cancer-specific survival (CSS) and overall survival (OS). The initial time for the time-to-event endpoints was defined as the date of the end radiotherapy.

Statistical analysis

The univariable analysis was performed using logistic regression (categorical) and log-rank test (time-dependent). For the main binary outcomes, variables with p<0.25 were incorporated in the multivariable logistic regression model. Due to the reduced number of events and the fact that DTI did not affect time-dependent outcomes (as shown in the section "Disease control and survival"), a multivariate analysis (Cox regression model) of the survival outcomes was not performed. The time-dependent endpoints were analyzed via the Kaplan-Meier method [13], with patient death included as a censoring event for LRC and MFS endpoints. All statistical analyses were performed using IBM SPSS Statistics, Build 1.0.0.1508, Armonk, NY, USA. A graphic representation of the logistic regression model was performed using Microsoft Excel v. 16.57.1 (Redmond, WA).

## Results

Patients and treatment characteristics

The median age of the cohort was 60.6 years (IQR 53.1-69.1). The most common symptoms at presentation were rectal bleeding (79.2%), rectal pain (14.9%), and constipation (10.9%). Around 56% were male and 17.8% were active smokers at the time of diagnosis and 25% of the females (11/44) had hysterectomy prior to the diagnosis of rectal cancer for benign causes. The median initial carcinoembryonic antigen (CEA) was 3.2 ng/mL (IQR: 1.9-7.5) and 51.5% of patients were stage III.

The majority of patients (82.2%) were treated with 3D conformal radiotherapy (3DCRT) and 17.8% were treated with intensity-modulated radiation therapy (IMRT) or volumetric modulated arc therapy (VMAT); 97% of the cases received concurrent fluoropyrimidine-based chemotherapy. The surgery involved abdominal perineal resection (APR) in 47.5% of the cases. Minimally invasive surgery (MIS) was used in 56.4% of the patients. Additional patient and treatment characteristics were presented in Table 1.

**Table 1:** Patient and treatment characteristics. IQR: interquartile range. BMI: body mass index. CEA: carcinoembryonic antigen. AV: anal verge. AJCC American Joint Committee on Cancer. pCR: pathological complete response. RT: radiotherapy. 3DCRT: 3D-conformal radiotherapy. IMRT: intensity-modulated radiation therapy. VMAT: volumetric modulated arc therapy. MV: megavolt. MIS: minimally invasive surgery. LAR: low anterior resection. APR: abdominal perineal resection. NA: not available.

Characteristic	Overall N (%)	Median (mean)	IQR (25-75%)
Age (years)	101	60.6 (60.8)	53.1-69.1
Sex			
Male	57 (56.4)	-	-
Female	44 (43.6)	-	-
BMI (kg/m^2^)	99	27.7 (28.5)	24.4-31.2
Smoking			
non-current (never or former)	83 (82.2)	-	-
current	18 (17.8)	-	-
Initial CEA (ng/mL)	92	3.2 (8.1)	1.9-7.5
Distance from AV (cm)	100	6.0 (6.2)	3.0-9.0
Initial Tumor Size (cm)	86	4.8 (4.9)	3.0-6.0
Clinical AJCC stage			
II	48 (47.5)	-	-
III	52 (51.5)	-	-
NA	1 (1.0)	-	-
RT Technique			
3DCRT	83 (82.2)	-	-
IMRT/VMAT	18 (17.8)	-	-
Energy			
6 MV	11 (10.9)	-	-
15 MV	90 (89.1)	-	-
RT timing			
AM	67 (66.3)	-	-
PM	34 (22.7)	-	-
Time from RT to surgery (weeks)	101	7.1 (7.6)	6.1-8.9
Type of Surgery			
open	40 (39.6)	-	-
MIS – laparoscopic or robotic	57 (56.4)	-	-
NA	4 (4.0)	-	-
Surgical Procedure			
LAR	53 (52.5)	-	-
APR	48 (47.5)	-	-
Margins			
negative	89 (88.1)	-	-
close/positive	12 (11.9)	-	-
Lymphadenectomy			
nodes removed	101	14.0 (15.1)	11.0-18.0
ypN0	69 (68.4)	-	-
ypN+	32 (31.6)	-	-
Pathological AJCC stage			
complete response (pCR)	18 (17.8)	-	-
ypI	23 (22.8)	-	-
ypII	28 (27.7)	-	-
ypIII	32 (31.7)	-	-
Adjuvant chemotherapy			
no	31 (30.7)	-	-
yes	59 (58.4)	-	-
NA	11 (10.9)	-	-

Acute toxicity and treatment interruptions

**Figure 1:**
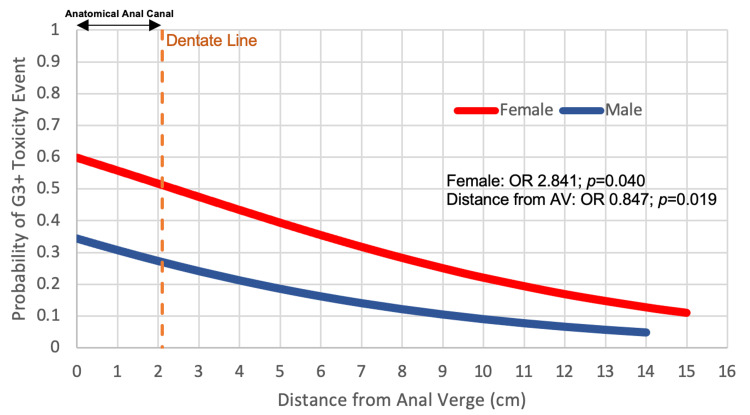
Factors affecting the probability of grade 3+ (G3+) toxicity event. Sex (male (reference)/female; odds ratio 2.841, p=0.040) and distance from the AV (continuous centimeters proximal from AV; odds ratio 0.847; p=0.019). Dentate line: 2.1 cm proximal from AV (dashed orange line).

The incidences of grade 0, 1, 2, 3, and 4 toxicities were: 2.1%, 41.0%, 30.5%, 25.3%, and 1.1%, respectively. The most common G3+ toxicities were peri-anal dermatitis (14.7%) and diarrhea (7.4%). The G3+ events were more frequent in females (OR 2.84, p=0.040) and patients with tumors close to the anal verge (AV) (continuous, each centimeter from AV to proximal rectum: OR 0.85, p=0.019, Figure 1).

Additionally, 11 cases (10.9%) developed DTI which occurred before or at fraction number 25 in five patients (45%) and during the boost phase (before fractions 26, 27 or 28) in 6 cases (55%). One patient had a bowel perforation after fraction number 17 of the pelvic field, requiring immediate surgery.

Table 2 describes the grade 2+ events that each patient who developed DTI experienced during RT. In this subgroup, the most frequent G3+ toxicities were diarrhea (45.5%), peri-anal dermatitis (18.2%), and weight loss (18.2%). The only variable associated with DTI was G3+ toxicity (OR 50.00, p<0.001, Table 3). Among female patients, the previous hysterectomy was not associated with grade 3+ toxicity (12/32 vs. 3/11, p=0.541).

**Table 2:** Cases with DTI and related toxicity. RT: radiotherapy. G3+: grade 3 or greater toxicity. DTI: definitive treatment interruption.

Case	RT Fractions (delivered/total)	Maximal Toxicity	Diarrhea	Peri-Anal Dermatitis	Weight Loss	Bowel Perforation	Rectal Pain	Emesis	Fatigue	Nausea	Dysuria
1	17/28	4	1	2	1	4	2	0	1	0	0
2	26/28	3	2	0	0	0	3	0	2	2	2
3	25/28	3	3	0	0	0	0	0	1	0	0
4	26/28	3	3	0	0	0	2	0	0	0	2
5	27/28	3	3	0	0	0	1	0	2	2	0
6	27/28	3	3	0	1	0	0	1	1	1	0
7	23/28	3	0	0	3	0	0	3	2	2	0
8	26/28	3	2	3	0	0	0	0	2	1	0
9	25/28	3	3	0	3	0	0	0	0	0	0
10	23/28	2	2	0	0	0	0	0	0	0	1
11	27/28	3	2	3	1	0	0	0	0	0	0

**Table 3:** Logistic regression analyses. CI: confidence interval. AV: anal verge. DTI: definitive treatment interruption. pCR: pathological complete response. cT: clinical tumor. RT: radiotherapy. AJCC: American Joint Committee on Cancer. * multivariable logistic regression; ** univariate logistic regression; *** ypI-II: stages ypI and ypII grouped

Endpoint	Variable	Category	Odds Ratio (95% CI)	p-value
G3+ toxicity (n=94) *	Sex	Male	Reference	
		Female	2.841 (1.050-7.692)	0.040
	Distance to AV	Continuous	0.847 (0.737-0.974)	0.019
DTI (n=95) **	G3+ toxicity	No	Reference	
		Yes	50.000 (5.917-422.520)	<0.001
pCR (n=85) *	cT size	Continuous	0.460 (0.280-0.756)	0.002
	Distance to AV	Continuous	1.249 (1.014-1.538)	0.036
	Time RT to surgery	< 6 weeks	Reference	
		≥ 6 weeks	9.144 (0.851-98.310)	0.068
Definitive Ostomy **	Distance to AV	Continuous	0.647 (0.531-0.787)	<0.001
	yp AJCC Stage	ypI-II*	Reference	
		ypIII	2.939 (0.859-10.060)	0.086

Pathological response and ostomy

Surgical resection occurred after a median of 7.1 weeks (IQR: 6.1-8.9) from the end of neoadjuvant therapy and consisted of total meso-rectal excision. Relatively to the initial clinical AJCC group stage, 57.4% had downstaging (including 17.8% pCR), 25.7% had no change in stage, and 15.8% had a more advanced disease in the final pathology report. Eighty-eight percent achieved negative margins. The dOST rate was 56.4% (57/101). DTI events did not impact pCR rate (OR 0.43, p=0.435) or dOST (OR 1.40, p=0.611) (Table 3).

Disease control and survival

**Figure 2:**
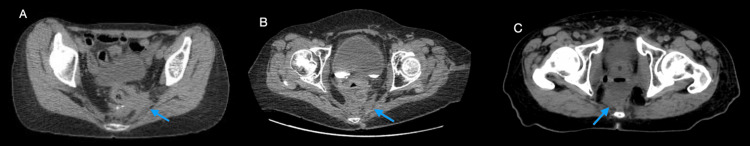
Axial computed tomography slices of the local failure events. Case A: 52-year-old female. cT3cN0/LAR/ypT3ypN2a, negative margins. Isolated local recurrence after 77.7 months retreated with neoadjuvant chemotherapy and resection. Last status: alive with no evidence of disease (125.8 months). Case B: 53-year-old female. cT3cN2b/APR/ypT3ypN2a, positive margin (distal). Local recurrence after 14.4 months with widespread distant failure before. Last status: cancer-related death (15.4 months) Case C: 55-year-old male. cT3cN1/LAR/ypT4bypN2b, positive margin (distal) and close margin (radial). Local recurrence after 11.1 months with widespread distant failure before. Treated with palliative radiotherapy 30 Gy (10 x 3.0Gy). Last status: cancer-related death (19.4 months).

**Figure 3:**
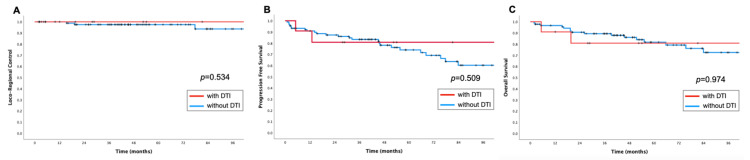
Kaplan-Meier representation of survival outcomes. A: Loco-regional control. B: Progression-free survival. C: Overall survival. Univariate (log-rank test). Blue line: cases without definitive treatment interruption (DTI). Red line: cases with DTI.

After 49.5 months median (mean 56.7 months) follow-up interval, three patients developed loco-regional failure (3y/5y LRC 97.8%/94.4%) (Figure 2A-2C). DTI event was not associated with LRC (5y 100% with DTI vs. 97.5% without DTI, p=0.534) (Figure 3A). The only factors associated with local failure were close/positive margins (5y LRC 76.2% vs. 100% for negative margins, p<0.001) and pathologic stage ypIII (5y LRC 92.3% vs. 100% for pCR/stage ypI/stage ypII, p=0.002).

Additionally, 19 patients developed distant metastasis, which more commonly involved non-regional lymph nodes (12/19 = 63.1%); lung (10/19 = 52.6%), and liver (9/19 = 47.4%). The 3y/5y MFS, PFS, CSS and OS were: 85.8%/78.9%, 83.0%/74.7%, 90.3%/85.2% and 88.4%/81.6%, respectively. DTI events were not associated with MFS (p=0.946), PFS (p=0.509), CSS (p=0.584) or OS (p=0.974) (Figures 3B-3C).

## Discussion

To our knowledge, the present study is the first to focus on the events of definitive interruption of RT delivery in the neoadjuvant treatment of LARC. We found that one in every 10 patients treated with long-course chemoradiotherapy presented DTI and that these events were strongly associated with G3+ toxicity. Although these unexpected interruptions did not impact oncologic outcomes (including pathological response, the need for definitive ostomy, and local control), they indicate that improvements in treatment tolerability are necessary.

Grade 3 diarrhea can be defined as seven or more stools per day over baseline limiting self-care activities of daily living [12]. In our cohort, these events occurred in less than 10% of the total cases; however, they preceded almost 50% of the DTI events. The secondary dehydration and reduction in performance status due to this side effect play an important role in the decision to suspend treatment. Intriguingly, female patients experienced higher rates of grade 3+ toxicity. A similar association with sex was previously described by a group from the University of Calgary [14] which hypothesized that the anatomical changes of hysterectomized patients (four of 11 women in their series) were responsible for such an increase in toxicity by exposing more small bowel in the treatment field. In our study, however, no association between the absence of the uterus at the time of RT and toxicity (p=0.541) was noted. In addition to that, tumors close to the AV were associated with more toxicity, indicating that a better understanding of the dose constraints for organs at risk in the lower pelvis [14,15] and of the gastrointestinal physiology [16-17] have the potential to improve tolerability. 

Despite having a reduction in the RT dose delivered, cases with DTI did not have worse oncologic outcomes, including pCR and local control. This observation indicates that doses higher than 45 Gy may not be necessary for LCRT. In that respect, researchers from Toronto (Canada) previously compared the results of three phase II studies that used 40 Gy, 46 Gy, and 50 Gy in the neoadjuvant setting [18]. They found a higher pCR rate with increasing the dose (15% vs. 23% vs. 33%, respectively, p=0.07), but no improvement in 2y-local recurrence-free survival above 46 Gy (72% vs. 90% vs. 89%, p=0.02). Of note, the high rate of local recurrences reported may indicate that TME was not performed for all cases and possibly more utilized in the later protocols with high doses, disfavoring the lower dose arm. More recently, some attempts to further escalate the neoadjuvant RT dose with TME were performed. One phase III study from Denmark and Canada [19] compared two radiation doses (EQD2 49.6 Gy10 vs. 62.1 Gy10) showing the same pCR in both arms (18% vs. 18%). Similarly, the RECTAL-BOOST trial conducted in the Netherlands [20] failed to improve the pathological complete response (for operable cases) or two-year sustained clinical response (for watch-and-wait cases) with an escalated boost delivering an EQD2 of 66.3 Gy10 (compared to EQD2 50 Gy10).

A comparison between the prescription doses of SCRT and LCRT gives another relevant perspective. Applying the normalization by the equivalent dose in 2 Gy fractions (EQD2) and assuming α/β=10, the LCRT regimen of 45 Gy in five weeks (25 x 1.8Gy, EQD2 44.2Gy10) delivers 40% more dose when compared with SCRT (5 x 5 Gy, EQD2 31.2 Gy10). This increase raises to 60% when using the fractionation of 50 Gy in five weeks (25 x 2Gy, EQD2 50 Gy10) or adding a 5.4 Gy boost (28 x 1.8 Gy, EQD2 49.6 Gy10). Despite higher doses in LCRT, the local control of both LCRT and SCRT strategies are similar [21].

Cases with close or positive resection margins after TME had significantly worse loco-regional control when compared with patients with negative margins (five-year: 76.2% vs. 100%, p<0.001). Based on that and the fact the pre-operative boost did not improve local control, it could be hypothesized that omitting the pre-operative boost in order to leave room for additional radiation dose intra- [22] or post-operatively [23] could potentially benefit the subgroup of patients that failed to achieve clear margins [24-26] or at the time of a local recurrence [27]. The post-operative indication of boost has an enticing prospect, especially if no further therapy is planned after resection, which is the case in the total neoadjuvant therapy strategy [28-30]. For patients with tumors involving the anatomical anal canal (at or below the dentate line) [11], the risk/benefit ratio is even more unfavorable regarding the pre-operative boost. These patients have a higher risk of grade 3+ toxicity and no benefit of sphincter preservation since they require abdominal perineal resection and colostomy, independently of the theoretical additional downstaging effect of the boost. Importantly, tailoring the indication of boost based on pathological findings could spare unnecessary boost for patients with clear margins (88% in our cohort), automatically reducing the incidence of DTI by about half (55% of cases had interruption during the boost phase).

The limitations of the present study include its retrospective design and relatively modest sample size, which precluded a more robust multivariate analysis of the secondary survival outcomes. In addition, no data on chemotherapy tolerability (dose reduction events and the total number of cycles delivered) and patient-reported outcomes were available, which could have provided more comprehensive insights regarding treatment tolerability. Importantly, the findings of the present study do not apply to the scenario of organ preservation where radiation is potentially the only local treatment.

## Conclusions

In summary, we found that one in every 10 patients with LARC treated with LCRT presented a DTI event, which was strongly related to grade 3+ toxicity. These treatment interruptions affected the delivery of the boost before surgery without affecting the pCR, definitive ostomy rates, and local control. In addition, patients with positive margins after surgery had worse local control and survival, raising the question of whether the boost should be reserved for those who failed to achieve negative margins and delivered after surgery.
